# Comparison of cardiovascular magnetic resonance to single-photon emission computed tomography in women with suspected coronary artery disease: a CE-MARC sub-study

**DOI:** 10.1186/1532-429X-15-S1-P194

**Published:** 2013-01-30

**Authors:** John P Greenwood, Manish Motwani, Neil Maredia, Julia Brown, Colin Everett, Jane Nixon, Petra Bijsterveld, Catherine J Dickinson, Sven Plein

**Affiliations:** 1 MCRC & LIGHT, University of Leeds, Leeds, UK; 2 CTRU, University of Leeds, Leeds, UK; 3 Nuclear Cardiology, Leeds General Infirmary, Leeds, UK

## Background

Coronary artery disease (CAD) is the leading cause of death in women and under-diagnosis contributes to their high mortality. Breast attenuation and smaller heart size can make myocardial perfusion imaging with SPECT particularly challenging in women.This study compared the gender-specific diagnostic performance of CMR and SPECT in the CE-MARC study.

## Methods

CE-MARC was a prospective study of 752 patients with suspected angina. All patients were scheduled for CMR, SPECT and X-ray coronary angiography. Multi-parametric CMR comprised adenosine stress/rest perfusion, cine imaging, late gadolinium enhancement and MR coronary angiography. Gated adenosine stress/rest SPECT was performed using 99mTc-tetrofosmin. The primary outcome was the diagnostic accuracy of multi-parametric CMR and SPECT to detect CAD in female and male subgroups. A secondary outcome was a comparison of the perfusion-only components of CMR and SPECT in both sexes according to LV mass and disease extent, and in females according to bra size.

## Results

**Figure 1:**
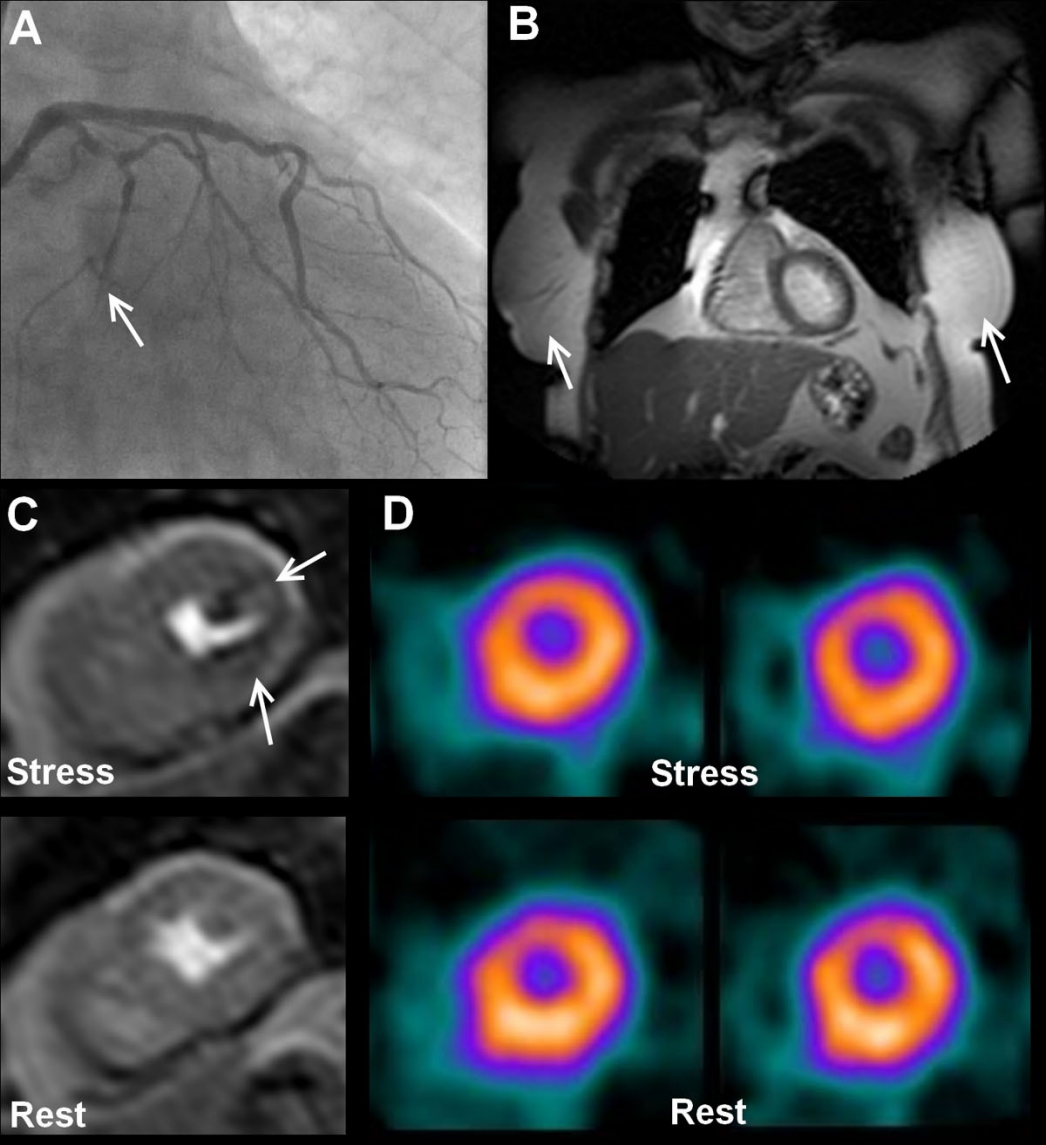
A 74 year old lady with previous PCI to the LAD artery had diffuse disease in the LCx artery with an occluded OM1 branch on X-ray angiography (arrow, A). Her breast size was average (bra-cup size C) as seen on CMR survey images (arrows, B). Left ventricular mass (65.4g) was within the lowest tertile for our population. Stress perfusion CMR identified subtle inducible ischemia in the inferolateral wall (arrows, C) concordant with the angiogram. However, SPECT did not identify any significant inducible ischemia (D). This case illustrates the difficulties of imaging subjects with small hearts (mostly females) with both perfusion techniques. Although subtle, CMR is still able to identify inducible ischemia as a result of its greater spatial resolution.

**Figure 2:**
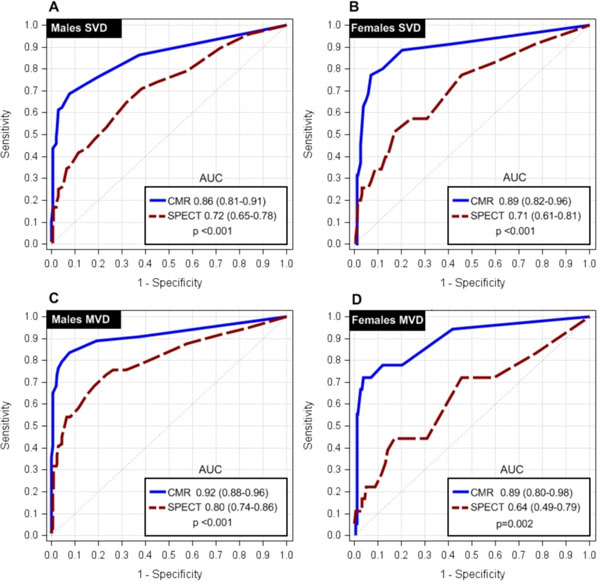
ROC curves generated using summed stress scores (n=393 males, 235 females). The diagnostic accuracy of stress perfusion CMR was significantly greater than SPECT in both single-vessel disease (SVD) and multi-vessel disease (MVD) - for both sexes (all p values <0.05). In SVD (A, B), the diagnostic accuracy was similar between males and females for both stress perfusion CMR (AUC, M: 0.86 vs. F: 0.89; p=0.420) and SPECT (AUC, M: 0.72 vs. F: 0.71; p=0.941). In MVD (C,D), stress perfusion CMR maintained a similar diagnostic accuracy between sexes (AUC, M: 0.92 vs. F: 0.89; p=0.635) but SPECT had a significantly lower diagnostic accuracy in females (AUC, M: 0.80 vs. F: 0.64; p=0.045). AUC = area under the curve. CMR = cardiovascular magnetic. SPECT= single-photon emission computed tomography.

235 females and 393 males had interpretable CMR, SPECT and X-ray angiography (Figure 1). For CMR, the sensitivity in both sexes was similar (88.7% vs. 85.6%, p=0.57), as was the specificity (83.5% vs. 82.8%, p=0.86). For SPECT, the sensitivity was significantly worse in females than males (50.9% vs. 70.8%, p<0.01), but specificities were similar (84.1% vs. 81.3%; p=0.48). Sensitivity in both female and male groups was significantly higher with CMR than SPECT (p<0.0001 for both) but specificity was similar (p>0.05 for both). On ROC analysis, perfusion CMR outperformed SPECT in females (AUC: 0.90 vs. 0.67, p<0.0001) and in males (AUC: 0.89 vs. 0.74, p<0.0001). With perfusion CMR the diagnostic accuracy was similar in both sexes (p=1.00), but with SPECT, it was significantly worse in females (p<0.0001). For perfusion CMR the diagnostic accuracy was similar in both sexes in both single-vessel and multivessel CAD. However, for SPECT the diagnostic accuracy was significantly lower in females with multivessel CAD (Figure 2).

## Conclusions

In both sexes, CMR has greater sensitivity than SPECT but similar specificity. Unlike SPECT, there are no significant gender differences in the diagnostic performance of CMR. The major challenge for SPECT in females appears to be the smaller heart size rather than breast attenuation artefacts. CMR may be less susceptible to these effects due to its inherent higher spatial resolution. These findings plus an absence of ionising radiation exposure, mean CMR should be considered the preferred non-invasive test for females with suspected CAD.

## Funding

CE-MARC was funded by the British Heart Foundation (BHF). JPG and SP receive an educational research grant from Philips Healthcare. SP is funded by a BHF fellowship (FS/1062/28409).

